# Forensic applications of the skin microbiome: a structured narrative review of donor association, contact inference, and crime-scene reconstruction

**DOI:** 10.3389/fmicb.2026.1947173

**Published:** 2026-09-18

**Authors:** Yating Song, Yuling Xing, Yang Liu, Xiang Zhang

**Affiliations:** 1 Law School, Qingdao University, Qingdao, Shandong, China; 2 Clinical Laboratory, Qingdao Hiser Hospital Affiliated with Qingdao University (Qingdao Traditional Chinese Medicine Hospital), Qingdao, China

**Keywords:** donor association, evidence interpretation, machine learning, skin microbiome, touch microbiome

## Abstract

**Background:**

Skin-associated microbial communities are individualized, anatomically structured, temporally dynamic, and readily transferred to contacted surfaces. These properties have generated interest in the skin microbiome as complementary forensic evidence when conventional human DNA or fingerprint evidence is limited.

**Methods:**

We conducted a structured narrative review guided by the Scale for the Assessment of Narrative Review Articles (SANRA). PubMed/MEDLINE was searched from database inception through 2 August 2026, and reference lists of eligible studies and recent reviews were examined. Peer-reviewed studies were evaluated for relevance to skin microbial individuality, temporal stability, touch transfer, donor or source inference, postmortem interval estimation, analytical standardization, and legal or ethical interpretation. Evidence was synthesized according to study realism, analytical resolution, validation design, and proximity to forensic casework.

**Results:**

The biological basis for forensic use is supported by reproducible inter-individual and body-site variation, including persistent strain- and gene-level features. Controlled studies show that skin-associated communities can be recovered from touched objects and clothing and can support donor differentiation, particularly when targeted markers, microbial single-nucleotide polymorphisms, or metagenomic features are used. Nevertheless, performance estimates are strongly affected by cohort size, body site, substrate, time since deposition, environmental background, contamination, and machine-learning design. Evidence for body-site and biological-trace source inference is promising, whereas postmortem interval estimation, chronological contact reconstruction, and spatial trajectory inference remain exploratory.

**Conclusion:**

Skin microbiome profiling should currently be regarded as a source of investigative intelligence and potentially complementary evidence rather than a replacement for human DNA profiling. Translation into practice requires harmonized protocols, realistic multicenter validation, open and representative reference datasets, explicit error-rate reporting, likelihood-based interpretation, and proportionate ethical and legal safeguards.

## 1 Introduction

Research-level donor identification and crime-scene reconstruction depend on analytical methods that can associate people, objects, locations, and events. Short tandem repeat (STR) profiling and fingerprint examination remain the principal tools for individualization because they offer high discriminatory power, validated laboratory workflows, and established statistical interpretation. Their performance, however, can be reduced when biological material is scarce, environmentally degraded, inhibited, or composed of multiple contributors. Brief contacts may produce incomplete human DNA profiles, and visible fingerprints may be absent or unsuitable for comparison. These limitations have encouraged investigation of complementary biomarkers that can recover information from low-biomass traces without displacing established evidence (Robinson et al., 2021; Gouello et al., 2021).

The human microbiome is one such candidate. Microorganisms and their nucleic acids are abundant on epithelial surfaces, and microbial communities vary across body sites, hosts, and environments. Skin is especially relevant because it is the body’s principal interface with clothing, personal belongings, architectural surfaces, and other people. The resulting microbial profile is shaped by stable host-associated features as well as recent exposures. During contact, part of this profile may be transferred to an object or another person, creating a trace that can be recovered even when human DNA is limited. Early work associating fingertip bacterial communities with computer keyboards established an influential proof of concept for microbiome-based forensic inference (Fierer et al., 2010). Subsequent metagenomic studies demonstrated that personal microbial features can persist over time and that strain- and gene-level information can provide greater discrimination than broad taxonomic abundance alone (Franzosa et al., 2015; Oh et al., 2016).

The expression “microbial fingerprint” is a useful pedagogical analogy but can also mislead; the term “microbial profile” or “signature” is more accurate for evidentiary contexts. A conventional fingerprint is a largely fixed anatomical pattern, whereas a skin microbial profile is a dynamic ecological measurement. It depends on the sampled body site, collection method, extraction chemistry, sequencing target, bioinformatic pipeline, environmental background, and time between questioned and reference sampling. A microbial association therefore cannot be interpreted as identity merely because a classifier assigns two samples to the same person. The evidentiary question is not only whether samples are similar, but how much more probable the observed similarity is under competing propositions about source and transfer. That distinction becomes crucial when moving from proof-of-concept classification to casework interpretation (Schmedes et al., 2018; Woerner et al., 2019; Sherier et al., 2021, 2022; Ruuskanen et al., 2022; Balacco et al., 2025; Knight et al., 2018; Sinha et al., 2017).

Technical development has progressed rapidly. Partial and full-length 16S rRNA sequencing are widely used for bacterial community profiling; internal transcribed spacer sequencing can assess fungi; shotgun metagenomics can resolve strains, genes, viruses, and other microbial components; and targeted panels can focus on informative clade-specific loci or microbial single-nucleotide polymorphisms (SNPs). Random forests, support vector machines, nearest-neighbor classifiers, and deep-learning architectures have all been applied to these data (Schmedes et al., 2018; Woerner et al., 2019; Sherier et al., 2021, 2022; Procopio et al., 2021, 2024; Wang et al., 2026; Yılmaz et al., 2024; Huang et al., 2024, 2025; Li et al., 2026). These methods have produced encouraging donor-classification results under controlled conditions, but reported accuracy is not directly comparable across studies because cohorts, sampling schedules, validation schemes, and class definitions differ. High accuracy in a small closed-set experiment does not establish a low random-match probability in an open forensic population.

Potential applications have also expanded beyond individual differentiation. Skin-to-object and skin-to-clothing studies examine whether microbial traces can indicate contact and persistence (Procopio et al., 2021, 2024; Wang et al., 2026). Body-site and biological-trace studies investigate whether a questioned profile is more consistent with a particular anatomical or specimen source (Huang et al., 2024, 2025). Postmortem research evaluates time-dependent microbial succession as a possible indicator of postmortem interval (PMI) (Iancu et al., 2023; Johnson et al., 2016; Metcalf, 2019; Belk et al., 2018; Zhang et al., 2021; Hu et al., 2024). More ambitious proposals seek to reconstruct the order of contacts, movements between environments, or activity sequences. These latter claims require special caution because a scene sample is an ecological mixture influenced by transfer, survival, cleaning, subsequent handling, and background contamination. The previous version of this manuscript summarized these developments in a broad, chapter-like narrative. In response to editorial concerns, the present article has been comprehensively restructured as a methodologically transparent narrative review. The aims are to: (i) explain the biological basis of skin microbial individuality and variability; (ii) critically compare sampling, sequencing, and computational approaches; (iii) evaluate the strength and limitations of evidence for donor association, contact inference, PMI estimation, and crime-scene reconstruction; and (iv) define the validation, ethical, and legal requirements that should be met before microbiome evidence is used operationally. Throughout the review, established observations are distinguished from emerging and exploratory applications.

## 2 Review methodology

### 2.1 Review design and reporting framework

This article was designed as a structured narrative review rather than a systematic review or meta-analysis. The evidence base comprises heterogeneous designs, including longitudinal human microbiome surveys, controlled touch-transfer experiments, targeted-marker development, machine-learning proof-of-concept studies, postmortem studies, methodological standardization research, and legal or ethical analyses. The outcomes are likewise heterogeneous and include ecological distances, classification accuracy, taxonomic or strain-level resolution, persistence, prediction error, and conceptual admissibility. A pooled quantitative estimate would therefore be neither statistically coherent nor clinically or forensically meaningful.

The conduct and reporting of the review were guided by SANRA, which emphasizes the importance of a clear justification, explicit objectives, transparent literature searching, appropriate referencing, scientific reasoning, and relevant endpoint data (Baethge et al., 2019). PRISMA 2020 was consulted as a general reference for transparency but is not claimed as the reporting framework because this review was not prospectively designed as an exhaustive systematic review of a narrowly formulated intervention, exposure, or diagnostic-accuracy question (Page et al., 2020). This distinction is stated explicitly to avoid presenting a structured narrative synthesis as a systematic review.

### 2.2 Information sources and search strategy

PubMed/MEDLINE was searched from database inception through 2 August 2026 as the primary database, given its comprehensive coverage of biomedical, microbiome, and forensic microbiology literature. Scopus and Web of Science were consulted selectively for cross-reference validation but were not systematically searched; this represents a limitation of the review and is acknowledged as such. Backward citation searching was performed using the reference lists of eligible primary studies and recent forensic microbiome reviews (Robinson et al., 2021; Gouello et al., 2021; Clarke et al., 2017; Franceschetti et al., 2024; Pan and Lv, 2026; Dass et al., 2026). The core search combined terms describing the specimen or exposure (“skin microbiome”, “skin microbiota”, “cutaneous microbiome”, “touch microbiome”, and “touched surface”) with forensic outcomes (“forensic”, “donor association”, “human identification”, “source attribution”, “contact transfer”, “body-site inference”, “postmortem interval”, and “crime scene”) and analytical approaches (“16S”, “metagenomic”, “single nucleotide polymorphism”, “targeted marker”, “machine learning”, and “likelihood ratio”). Topic-specific combinations were used to improve sensitivity. The complete PubMed strategy, search date, eligibility framework, and data-charting fields are provided in Supplementary Table S1.

The search was intended to identify the principal empirical and methodological literature needed for a critical overview rather than to guarantee exhaustive capture of every microbiome publication. Foundational studies were retained even when published before the recent expansion of the field, because they established concepts such as object-user association, personal metagenomic codes, and temporal stability (Fierer et al., 2010; Franzosa et al., 2015; Oh et al., 2016). Recent studies were prioritized when they introduced higher-resolution markers, realistic transfer scenarios, or explicit forensic applications (Procopio et al., 2021, 2024; Wang et al., 2026; Yılmaz et al., 2024; Huang et al., 2024, 2025; Li et al., 2026).

### 2.3 Eligibility criteria and study selection

Peer-reviewed human studies were eligible when they examined skin-associated microbial individuality, anatomical variation, temporal stability, transfer to surfaces or clothing, donor classification, targeted microbial markers, microbial SNPs, biological-trace or body-site inference, or postmortem skin microbiome dynamics. Methodological studies and authoritative reviews were included when they directly informed collection, low-biomass contamination control, sequencing, bioinformatics, statistical validation, ethics, or legal interpretation. Studies of built environments were included when they addressed human-environment microbial exchange relevant to transfer and source attribution (Lax et al., 2014). Animal or soil decomposition studies were considered only when they informed the development or limitations of PMI models and were clearly distinguished from direct human evidence.

Clinical dermatology articles without a clear contribution to forensic interpretation, reports limited to disease diagnosis, conference abstracts, non-peer-reviewed promotional claims, and publications lacking sufficient methodological detail were excluded. Screening was conducted in two stages: titles and abstracts were assessed against the review objectives, followed by full-text evaluation of potentially eligible publications. Thirty-five publications were retained for the final critical synthesis. These comprised 21 primary research studies (including controlled transfer experiments, longitudinal cohort studies, and PMI modeling), 9 methodological or analytical pipeline papers (addressing collection, sequencing, bioinformatics, or validation), 3 authoritative reviews that directly informed the review framework, and 2 legal/ethical analyses. Methodological papers were classified separately from empirical studies because they did not generate primary forensic outcome data. We prioritized relevance and methodological contribution in study selection. Since this review is narrative rather than exhaustive, we do not include a PRISMA flow diagram or pooled effect estimates.

### 2.4 Data charting and critical synthesis

For each retained study, the review considered the population and sample size, body site or substrate, sampling schedule, storage and extraction approach, sequencing or targeted assay, taxonomic or genetic resolution, model type, validation design, reported performance, and stated limitations. Particular attention was given to whether model evaluation separated donors, time points, sampling sessions, substrates, and laboratories between training and testing. Replicate leakage, batch effects, class imbalance, feature selection performed before test-set separation, and the absence of independent validation were treated as major risks of optimistic performance.

Evidence was organized into four pragmatic maturity levels. Proof-of-concept evidence shows that a signal exists under controlled conditions. Controlled validation evaluates pre-specified methods across repeated samples, time points, or substrates. External or temporal validation tests the method in donors, populations, periods, or laboratories not used during model development. Casework or field validation evaluates realistic mixtures, environmental backgrounds, chain-of-custody conditions, and competing propositions. No single numerical quality score was assigned because the literature is too heterogeneous for a validated common risk-of-bias instrument. Instead, limitations are described in the text and summarized in the evidence tables.

## 3 Biological basis of forensic skin microbiome analysis

### 3.1 Composition and anatomical distribution

The skin microbiome comprises bacteria, fungi, viruses, archaea, microbial genes, and mobile elements distributed across a physically and chemically heterogeneous organ. Bacterial communities are commonly dominated by members of Actinobacteria, Firmicutes, Proteobacteria, and Bacteroidota, with genera such as *Cutibacterium*, *Staphylococcus*, and *Corynebacterium* repeatedly observed. These broad taxonomic patterns are not unique to individuals, but their relative abundance, strain composition, gene content, and interaction with less abundant organisms create individualized profiles (Franzosa et al., 2015; Oh et al., 2016; Dass et al., 2026; Scharschmidt and Segre, 2025).

Local physiology is a major organizer of community structure. Sebaceous sites tend to support lipophilic *Cutibacterium* species; moist regions commonly contain *Corynebacterium* and *Staphylococcus*; and dry sites often exhibit greater taxonomic diversity. Sebum, hydration, pH, oxygen tension, follicular density, local immune activity, and exposure frequency all contribute to these patterns. Consequently, a trace can encode at least two overlapping signals: the anatomical site from which it originated and the person who contributed it. This creates both opportunity and confounding. Body-site information may assist biological-source inference, but a classifier trained on one anatomical location may fail when a questioned sample was deposited from another location (Oh et al., 2016; Huang et al., 2024; Dass et al., 2026; Scharschmidt and Segre, 2025).

The hand microbiome is particularly important because hands contact many objects and environments. It is also unusually dynamic. A hand sample may contain resident organisms, transient environmental taxa, material transferred from other body sites, and microorganisms acquired from recently touched surfaces. The resulting diversity can enhance discrimination in a closed experimental dataset, but it can also reduce temporal comparability. Models should therefore distinguish persistent donor-associated markers from transient exposure signatures rather than assuming that every abundant feature reflects individual identity (Figure 1).

**Figure 1:**
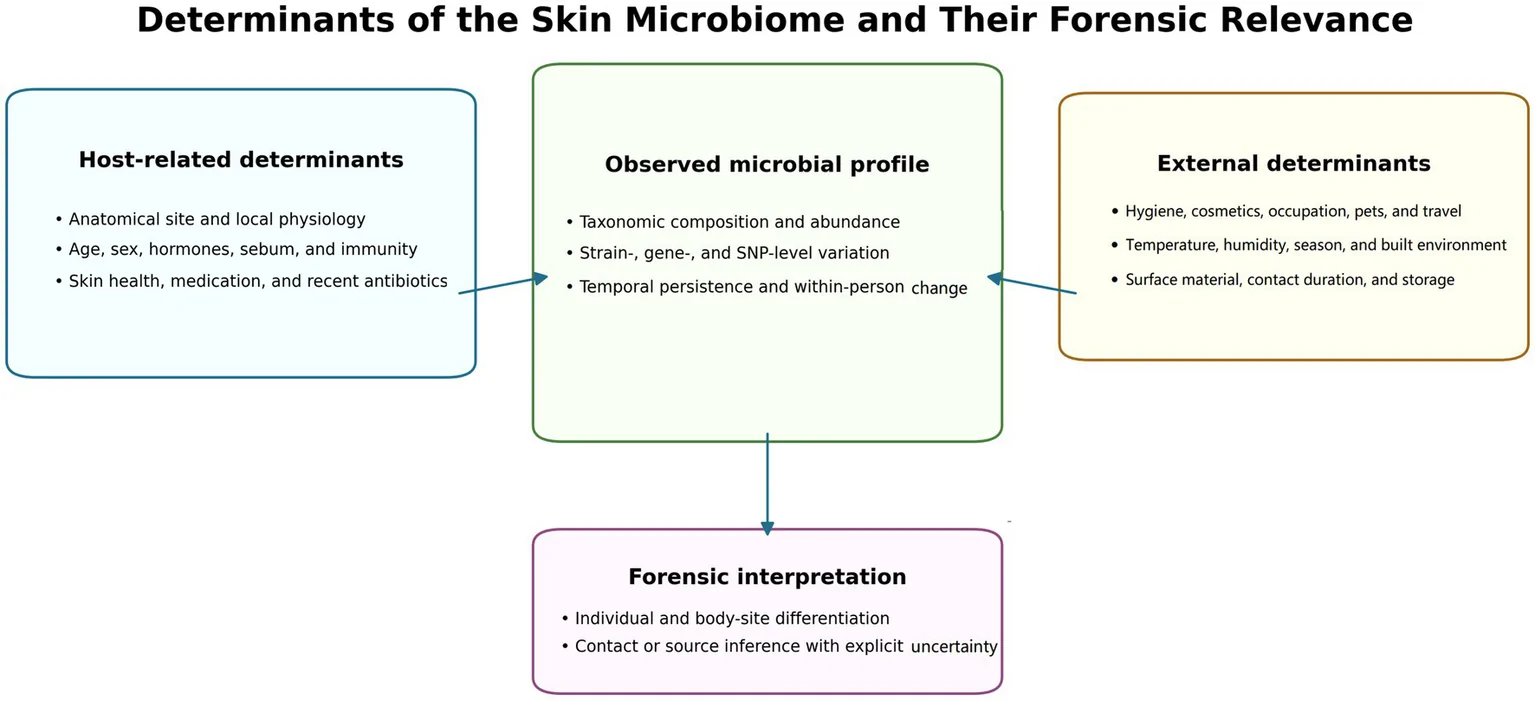
Intrinsic host factors and external exposures jointly shape the observed skin microbial profile. These determinants create individuality but also introduce uncertainty into forensic comparison. The figure is a conceptual schematic and does not contain experimental data.

Recent studies have extended beyond simple taxonomic cataloging to examine the co-occurrence patterns among skin microbes, that is, which taxa tend to be found together across individuals and body sites. These co-occurrence networks are non-random and reflect ecological interactions such as mutualism, competition, and niche sharing, as well as shared host and environmental drivers. Importantly, skin microbial communities can be classified into discrete community types or ecotypes, analogous to gut enterotypes, based on the relative abundance of dominant genera such as *Cutibacterium*, *Staphylococcus*, or *Corynebacterium* (Dass et al., 2026; Scharschmidt and Segre, 2025). These ecotypes are associated with host physiological traits (sebum production, hydration, age) and may influence how a community responds to transfer, environmental exposure, and post-mortem change. For forensic applications, co-occurrence structure and ecotype classification have two implications. First, an individual’s profile is not simply a list of taxa but a structured ecological network; similarity metrics that account for co-occurrence may be more informative than distance measures based on independent taxa. Second, ecotype membership may confound donor differentiation because individuals with similar physiological traits share similar community structures, reducing between-person variability. Conversely, ecotype information could assist body-site or biological-trace inference by providing a physiological context for the recovered sample. We have incorporated relevant literature on co-occurrence and skin ecotypes into this section (Clarke et al., 2017; Dass et al., 2026; Scharschmidt and Segre, 2025).

Fungal and viral components may offer additional information, yet the forensic literature remains dominated by bacterial markers. Internal transcribed spacer sequencing can characterize fungi, while shotgun metagenomics can recover bacteriophages, eukaryotic viruses, and gene-level features. In principle, combining microbial kingdoms may increase discrimination, especially when bacterial profiles converge. In practice, lower biomass, incomplete reference databases, and differing extraction requirements complicate multimicrobial analysis. Such approaches should be described as promising rather than mature until their added value is demonstrated in independent forensic datasets.

### 3.2 Inter-individual variation and strain-level individuality

Inter-individual variation provides the primary biological rationale for identification. Community-level studies show that people differ in the relative abundance and composition of skin taxa, even at matched anatomical sites. Metagenomic work further indicates that personal signatures are often stronger at strain, gene, or marker level than at genus level (Franzosa et al., 2015; Oh et al., 2016). This is biologically plausible because common species can contain highly diverse lineages, accessory genes, prophages, and mobile elements. A person may therefore share the same dominant species with many others while carrying a distinctive combination of variants within those species. Targeted forensic assays exploit this structure. Clade-specific markers and selected microbial SNPs reduce a complex metagenome to a panel of features that can be amplified and compared reproducibly (Schmedes et al., 2018; Woerner et al., 2019; Sherier et al., 2021, 2022). Such panels can be more practical than unrestricted metagenomic sequencing because they require less sequencing depth and simplify interpretation. The trade-off is that marker discovery may be population-specific. A panel developed in a limited cohort may select features associated with ancestry, geography, laboratory batch, or lifestyle rather than stable donor identity. Population-informative differentiation may be useful for some investigative questions, but it must not be confused with individualization (Figure 1).

Recent higher-resolution approaches, including full-length 16S sequencing, species-level profiling, microbial SNP analysis, and reduced-representation metagenomic strategies, have reported stronger discrimination than conventional short-amplicon community profiles (Wang et al., 2026; Huang et al., 2024, 2025; Li et al., 2026). These studies demonstrate that analytical resolution matters. They do not, by themselves, establish general forensic validity. The strongest design would show that a fixed marker set and locked analytical pipeline maintain performance across new donors, repeated sampling dates, body sites, environmental exposures, laboratories, and sequencing runs.

### 3.3 Host genetics, physiology, age, sex, and disease

Host factors influence the ecological background against which individual-specific variation is measured. Genetic variation can affect immune signaling, barrier function, lipid composition, and susceptibility to colonization. Age modifies sebum production, hydration, immune surveillance, and exposure patterns. Puberty commonly increases sebaceous activity, whereas aging can alter skin structure and microbial diversity. Sex-related hormonal and behavioral differences may also affect community composition. These factors contribute to individuality but are not fixed identifiers, and their effects may be weaker or less consistent than body-site and environmental influences (Dass et al., 2026; Scharschmidt and Segre, 2025).

Medication and dermatological disease can cause substantial departures from a person’s baseline. Topical antimicrobials, systemic antibiotics, acne treatment, inflammation, hospitalization, and altered barrier integrity may change taxonomic abundance or remove markers used by a classifier. A reference database constructed from healthy volunteers may therefore perform poorly in patients or in persons sampled after treatment. Forensic validation should include common perturbations or, at minimum, define the population to which results can be applied (Figure 1).

From a privacy perspective, the influence of physiology and disease is double-edged. These signals may improve source characterization, but deep microbial profiling can reveal information unrelated to the legal question, including medication exposure, health conditions, or cohabitation. Analytical pipelines should minimize collection and reporting of unnecessary sensitive information, particularly when shotgun metagenomics is used (McGuire et al., 2008).

### 3.4 Temporal stability, lifestyle, and environmental perturbation

A forensic identifier must retain enough stability for a questioned trace to be compared with a reference collected later. Longitudinal studies show that skin communities can preserve person-associated features over months, especially at strain or gene-content level, although relative abundances fluctuate (Franzosa et al., 2015; Oh et al., 2016). Stability is not uniform across body sites or taxa. Occluded and sebaceous locations may retain resident communities more consistently, whereas exposed hands are repeatedly perturbed by washing, objects, people, and outdoor environments.

Lifestyle and exposure contribute both noise and contextual information. Hygiene practices, cosmetics, occupation, pets, travel, household sharing, climate, season, temperature, humidity, and indoor or outdoor setting can alter the observed profile. Cohabiting people may exchange organisms or share environmental sources, increasing similarity without direct involvement in a specific event. Likewise, a distinctive environmental taxon on a questioned object may indicate the setting rather than the donor. These factors do not eliminate forensic utility, but they change the proposition being evaluated (Figure 1).

Temporal validation should therefore replicate the interval expected in casework. Splitting technical replicates or samples collected from one person during the same session between training and testing measures analytical repeatability, not temporal identification. More informative designs hold out later sampling sessions, altered hygiene conditions, seasonal periods, or independent environments. The resulting decline in performance is scientifically valuable because it defines the method’s operational window rather than merely maximizing accuracy.

### 3.5 Transferability, persistence, and ecological mixing

During contact, microorganisms can be transferred from skin to glass, plastic, metal, textile, mobile devices, keyboards, or other surfaces. The quantity and composition of the deposit depend on contact area, pressure, duration, friction, perspiration, donor shedding, and substrate properties. Pilot work has shown that microbial profiles can be recovered from touched surfaces when conventional STR profiles are incomplete, supporting their potential complementary value (Fierer et al., 2010; Procopio et al., 2021). Clothing experiments further demonstrate that donor, garment, environment, storage, and time all contribute to the recovered signal (Procopio et al., 2024).

Persistence should not be equated with direct contact. A profile detected days after deposition may reflect viable cells, extracellular DNA, environmental growth, or protected material within a porous substrate. Subsequent handling can add contributors, displace the earlier deposit, or selectively transfer abundant taxa. Cleaning may remove some organisms while selecting for others. In mixed samples, the most recent contactor will not necessarily dominate, and a strong microbial similarity does not establish that transfer occurred during the alleged event rather than through background or secondary transfer.

These issues are familiar in trace evidence but especially important for microbiomes because communities can change after deposition. Experimental transfer studies should separate deposition, persistence, secondary transfer, and growth. They should also include negative environmental controls and realistic competing donors. Without such controls, a model may simply learn the microbial ecology of a substrate or room rather than the identity of the person who touched it (Figure 1).

A more fundamental challenge is that transfer fundamentally alters the microbial environment. When skin microorganisms are deposited onto clothing, glass, or other substrates, they encounter different moisture, nutrient, oxygen, and UV conditions compared to their native skin habitat. This environmental shift can induce rapid compositional changes through differential survival, growth, or DNA degradation. Similarly, post-mortem changes, including cessation of immune activity, altered blood flow, and exposure to internal or environmental organisms, profoundly reshape the community. Consequently, a sample recovered from clothing or a cadaver represents an altered, mixed community rather than a faithful copy of the donor’s living skin profile. The defensible empirical comparison is therefore not between any two microbial profiles as if they were static identifiers, but rather between a questioned sample and a reference collected under conditions that approximate the transfer scenario, for example, matching a recent handprint on glass against a direct hand swab collected using the same substrate and deposition parameters. The consistent decline in classification performance when models are tested across different timepoints, distinct body sites, or various inanimate surfaces (Franzosa et al., 2015; Oh et al., 2016; Procopio et al., 2021, 2024; Wang et al., 2026) reflects this ecological reality: the skin microbiome is a context-dependent measurement, not a fixed biological barcode.

## 4 Analytical workflow and technological platforms

### 4.1 Scene sampling, reference sampling, and preservation

The pre-analytical stage is a major source of variation in low-biomass microbiome evidence. Swab material, wetting solution, sampled area, pressure, number and direction of strokes, anatomical site, substrate porosity, time since contact, storage temperature, and transport duration can alter both yield and community composition (Ruuskanen et al., 2022; Balacco et al., 2025). Unlike abundant clinical specimens, many forensic touch samples contain microbial biomass near the level of laboratory background. Small procedural differences can therefore create large proportional effects. Quantitative data on microbial biomass in forensic touch samples remain sparse. Published studies rarely report bacterial density per unit area, detection thresholds for specific substrates, or systematic comparisons of recovery efficiency across fabric types, surface materials, and storage conditions. Degradation kinetics, the rate at which microbial DNA becomes unamplifiable under different temperature, humidity, and substrate conditions, have not been characterized for most forensically relevant scenarios. Laundering effects on textile-associated microbial communities are similarly understudied (Procopio et al., 2024). This quantitative gap is a significant limitation: without such data, it is difficult to define the lower bound of reliable detection, to compare results across studies, or to establish whether a recovered signal exceeds background with statistical confidence. We acknowledge this gap explicitly and identify it as a priority for future method development.

A validated protocol should specify the collection device and area and should record scene metadata such as temperature, humidity, surface material, cleaning history, visible moisture, known handling, and storage conditions. Field blanks are necessary to measure contamination introduced by the swab, packaging, investigator, and collection environment. Reference samples should be collected using methods compatible with the questioned sample, ideally from the anatomically relevant site and at a documented interval after the event. When destructive sampling is unavoidable, the protocol should consider the needs of conventional DNA, fingerprints, and other trace examinations so that microbiome analysis does not compromise higher-priority evidence (Figure 2; Tables 1, 2).

**Figure 2:**
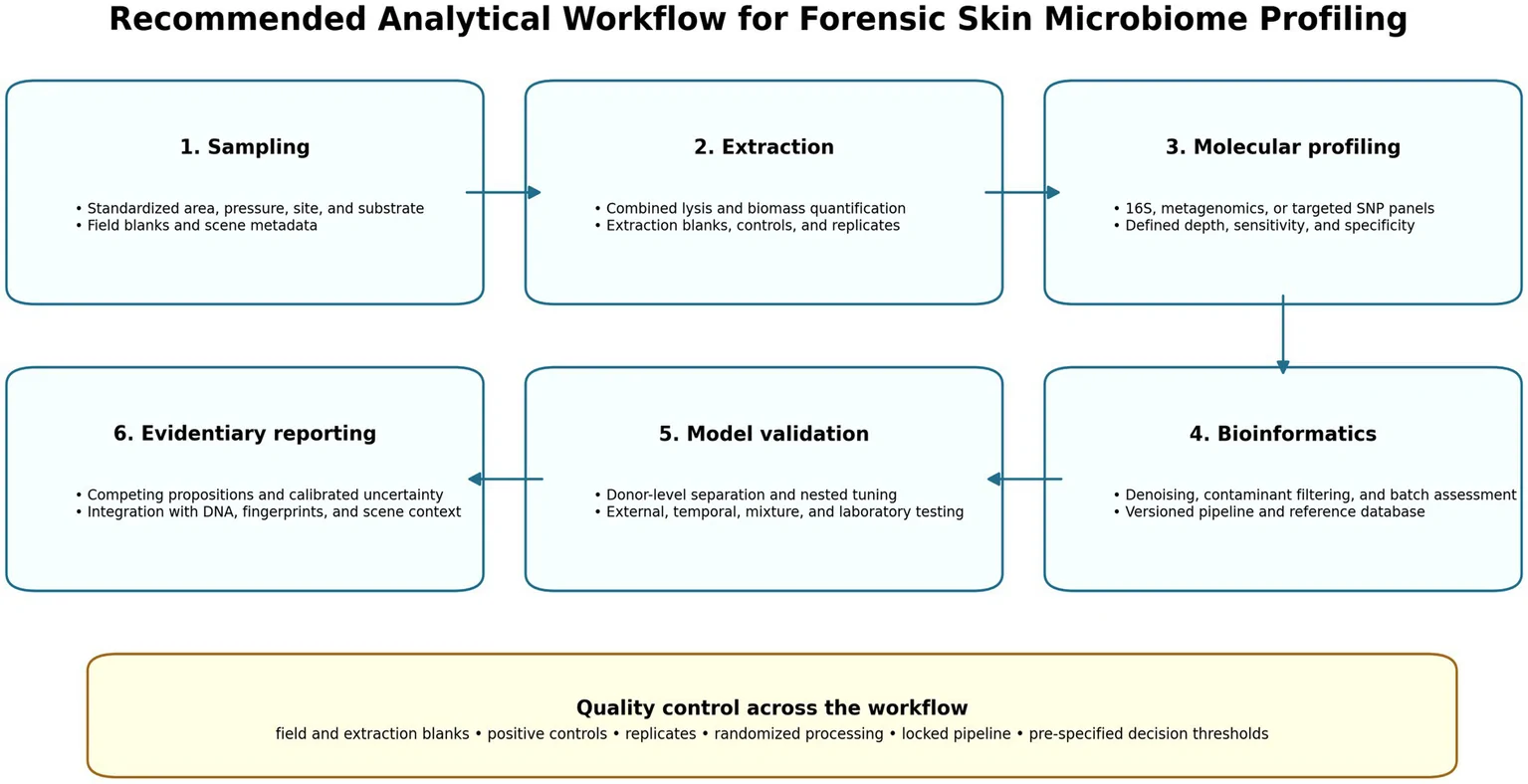
Recommended workflow for forensic skin microbiome profiling, from scene sampling and contamination controls to molecular profiling, validation, and qualified interpretation. The figure is a conceptual schematic and does not contain experimental data.

**Table 1:** Representative studies underpinning major forensic skin microbiome applications.

Design/application	Principal contribution	Important limitation	References
Fingertip and keyboard bacterial communities	Early proof-of-concept association between users and touched objects	Small controlled cohort; community-level 16S resolution	Fierer et al. (2010)
Longitudinal personal metagenomes	Demonstrated persistent personal metagenomic features	Not designed as a forensic casework validation	Franzosa et al. (2015)
Longitudinal skin microbiome	Established temporal persistence of strain-level skin features	Body-site and participant-specific; controlled sampling	Oh et al. (2016)
Targeted clade-specific skin markers	Developed a forensic-oriented targeted marker strategy	Population and laboratory transportability require validation	Schmedes et al. (2018)
Nearest-neighbor donor classification	Demonstrated donor assignment with targeted microbial markers	Closed-set classification is not a random-match probability	Woerner et al. (2019)
Population-informative markers and microbial SNPs	Improved resolution using selected genetic variants	Marker frequencies and temporal stability need broader estimation	Sherier et al. (2021, 2022)
Touch deposits on glass	Recovered microbial profiles from many touch samples, including samples with limited STR information	Pilot study with few volunteers and one principal surface type	Procopio et al. (2021)
Skin-to-clothing transfer and persistence	Demonstrated donor, garment, environmental, and temporal effects	Small cohort; environmental contribution complicates source attribution	Procopio et al. (2024)
Full-length and variable-region 16S sequencing	Improved characterization of skin and touched bacterial communities	Single-locus amplicon data remain below whole-metagenome strain resolution	Wang et al. (2026)
Body-site, fluid-source, species, and SNP profiling	Supported higher-resolution source inference and donor differentiation	External population, mixture, and interlaboratory validation remain limited	Huang et al. (2024, 2025)
2bRAD-M and hierarchical attention network	Illustrated high-resolution features with deep learning	Proof of concept; complex model at risk of overfitting without external validation	Li et al. (2026)
Postmortem skin microbiome	Demonstrated time-associated postmortem microbial change	Limited human cohorts; environment and cause of death remain confounders	Iancu et al. (2023), Johnson et al. (2016)

**Table 2:** Comparison of principal analytical approaches.

Approach	Main strength	Main limitation	Most defensible current role
Short 16S rRNA amplicon sequencing	Low cost, mature workflows, broad bacterial community profiling	Primer and region bias; limited strain resolution; compositional abundance	Ecological comparison, screening, body-site research
Full-length 16S rRNA sequencing	Improved taxonomic assignment and cross-region coverage	Still a single bacterial locus; platform and database effects	Enhanced community characterization and emerging source inference
Fungal ITS sequencing	Adds fungal community information	Variable length, low biomass, incomplete references	Complementary environmental or source research
Shotgun metagenomics	Strain, gene, virus, and functional resolution	Cost, human DNA, privacy, computational burden	Research-grade high-resolution donor and source analysis
Targeted clade/SNP panels	Fixed feature space, sensitivity, reproducibility, easier validation	Discovery-cohort dependence and marker dropout	Most practical candidate for future validated donor association
Reduced-representation metagenomics/k-mers	High discrimination with less sequencing than shotgun	Method-specific bias and limited external validation	Emerging proof-of-concept individual differentiation
Machine learning/deep learning	Captures high-dimensional interactions	Leakage, overfitting, poor calibration, limited interpretability	Classification research only unless externally validated and calibrated

Preservation strategies should prevent differential growth and DNA degradation. Refrigeration or freezing may be appropriate when available, but realistic casework often involves delays. Dry storage can reduce growth, whereas transport media may improve recovery but introduce reagent-associated background. Validation should compare the conditions likely to occur in the relevant jurisdiction rather than relying only on optimal laboratory storage (Figure 2; Tables 1, 2).

### 4.2 DNA extraction and contamination control

Skin communities contain Gram-positive and Gram-negative bacteria, fungi, and microbial structures with different resistance to lysis. Protocols based only on enzymatic or chemical treatment can under-represent organisms with thick cell walls. Combined enzymatic lysis and bead beating generally improve breadth, but aggressive disruption may fragment DNA and increase the release of human DNA. Extraction choice should therefore be linked to the downstream assay: amplicon sequencing, targeted SNP typing, full-length sequencing, and shotgun metagenomics have different requirements (Figure 2; Tables 1, 2).

Negative controls are indispensable. Extraction blanks, PCR blanks, and environmental controls should be processed in parallel and sequenced deeply enough to detect background features. Positive controls and reference materials help monitor lysis, amplification, and run-to-run performance. Samples and controls should be randomized across extraction and sequencing batches. Comparison of negative-control prevalence and abundance with sample profiles, along with replicate extraction and batch-level visualization, can help distinguish genuine low-abundance taxa from reagent contaminants. Biomass quantification, while useful for assessing whether a sample meets detection thresholds, does not by itself discriminate between authentic signals and background contamination (Ruuskanen et al., 2022; Balacco et al., 2025; Knight et al., 2018; Sinha et al., 2017).

Contamination cannot be solved with a universal blacklist. Some common reagent organisms may also be genuine skin or environmental taxa. Prevalence- and frequency-based approaches should therefore be combined with biological plausibility, negative-control abundance, and batch visualization. The contaminant-removal procedure must be defined before classification whenever possible, because tuning it to improve case labels risks information leakage (Figure 2).

It must be acknowledged that procedural blanks and negative controls, while essential, address only known and measurable contamination introduced during collection and laboratory processing. They do not resolve the more intractable problem that background environmental microbes, secondary transfer through intermediate objects or persons, or the recipient surface’s own resident ecology can produce microbial similarity that is indistinguishable from direct donor deposition. With current methods, contamination, in the broad sense of any microbial input not originating from the alleged donor via the alleged contact, cannot be definitively ruled out as the driver of an observed similarity. A statistical match, whether based on ecological distance or machine-learning classification, is therefore not proof of identity; it is an association that must be evaluated in light of competing explanations, including environmental background, shared exposure, and alternative transfer pathways.

### 4.3 Amplicon sequencing

Short-read 16S rRNA amplicon sequencing remains the most accessible approach for skin community surveys. It is economical, supported by mature denoising pipelines, and suitable for comparing many samples. Its limitations are equally important. Different hypervariable regions can produce different taxonomic profiles, primer bias can exclude informative organisms, copy-number variation distorts abundance, and many reads cannot be resolved below genus or species level. Consequently, short-amplicon profiles may describe ecological similarity without capturing the strain-level variation needed for individualization (Ruuskanen et al., 2022; Sinha et al., 2017; Wang et al., 2026).

Full-length 16S sequencing can improve taxonomic assignment and comparison across variable regions. Long-read platforms also reduce the need to infer a species from a short fragment. Nevertheless, full-length 16S remains a single-locus bacterial assay, and closely related strains may share identical sequences. Sequencing errors, platform-specific bias, and reference-database limitations must be considered. Its principal current value may be improved community characterization and body-site inference rather than definitive donor identification (Wang et al., 2026).

Fungal ITS sequencing expands the marker space and may be useful on surfaces or in environments where fungi persist differently from bacteria. However, fungal biomass is often low, ITS length varies, and reference annotations are incomplete. Multikingdom assays should demonstrate that the additional marker class improves calibrated forensic performance rather than merely increasing dimensionality (Figure 2).

### 4.4 Shotgun metagenomics and high-resolution markers

Shotgun metagenomics captures microbial genomes without targeting a single locus. It can resolve strains, accessory genes, viruses, plasmids, and functional pathways and has supported the concept of personal metagenomic codes (Franzosa et al., 2015). This resolution is attractive for forensic comparison because strain-level variation may remain individualized even when broad community composition changes. The approach also permits k-mer or marker-based analysis without relying entirely on taxonomic classification.

The main barriers are cost, low microbial biomass, high proportions of human DNA, computational complexity, and privacy. Host DNA depletion can improve microbial coverage but may introduce bias or consume limited evidence. Deep sequencing of a touch sample can also recover human genetic information or health-associated microbial features. Laboratories need clear policies for host-read handling, data retention, secondary analysis, and disclosure (McGuire et al., 2008). Targeted panels offer an alternative. The hidSkinPlex strategy and subsequent marker or SNP panels focus sequencing on informative microbial loci (Schmedes et al., 2018; Woerner et al., 2019; Sherier et al., 2021, 2022). This can improve sensitivity and reproducibility while reducing irrelevant information. Targeted assays are also more compatible with quality thresholds, allele or marker dropout modeling, and fixed interpretation software. Their validity, however, depends on stable performance outside the discovery cohort. Marker frequencies and within-person variability must be characterized in diverse populations, and the assay must tolerate low template, mixtures, inhibition, and temporal change (Figure 2; Tables 1, 2).

Recent studies have combined species-level profiles, microbial SNPs, reduced-representation metagenomics, and hierarchical attention networks (Huang et al., 2024, 2025; Li et al., 2026). These developments show that higher-resolution features and more flexible models can improve classification in research datasets. They also increase the risk of overfitting. A complex model is not inherently more forensically informative than a simpler targeted assay unless it is externally validated, calibrated, and interpretable.

### 4.5 Bioinformatics, ecological distances, and machine learning

Microbiome data are sparse, compositional, and high dimensional. Relative abundance is constrained to sum to one, so an apparent increase in one taxon can result from a decrease in another. Bray–Curtis dissimilarity describes abundance-based differences, whereas UniFrac incorporates phylogenetic relationships. These distances are useful for visualization and exploratory clustering, but they are not probabilities of common source. The choice of transformation, zero handling, feature filtering, and normalization can substantially change the result (Knight et al., 2018).

Supervised classifiers, including random forests, support vector machines, nearest-neighbor models, and deep neural networks, can exploit multivariate patterns that are not apparent from individual taxa. Their evaluation must mirror the forensic question. Samples from one donor should not be divided across training and test sets when the goal is to identify unseen donors or later samples. Feature selection, normalization, and hyperparameter tuning must be performed within the training folds. Nested cross-validation or a completely independent test set is preferable to a single random split. Overall accuracy can conceal important errors. Class imbalance may produce high accuracy while minority donors or sources are poorly recognized. Relevant outputs include sensitivity, specificity, false-match rate, false-non-match rate, precision, recall, calibration, and performance as the reference database grows. Open-set testing is particularly important because the true contributor may not be represented in the database. A model should be permitted to return an inconclusive or unknown result rather than forcing every questioned profile to the nearest donor (Figure 2; Tables 1, 2).

Batch effects require explicit examination. If all samples from one donor are processed together, a classifier may learn extraction date, plate, reagent lot, or sequencing run. Randomized laboratory processing and external laboratory validation are therefore part of model validity, not merely good laboratory practice. Versioned code, locked reference databases, and documented software dependencies are also essential for reproducibility (Ruuskanen et al., 2022; Knight et al., 2018; Sinha et al., 2017).

### 4.6 Statistical interpretation and likelihood ratios

A donor classifier answers a different question from a forensic likelihood ratio. Closed-set classification asks which known class is most similar to the sample. A likelihood ratio compares the probability of the observations under two propositions, for example that the questioned trace originated from the person of interest versus an unknown alternative person from a relevant population. This requires estimates of within-source variation, between-source variation, measurement error, and the effects of time and transfer.

Microbiomes complicate these estimates because the source is not a static genotype. The reference profile may differ from the profile at deposition, and the deposited material can change on the substrate. Relevant propositions may concern source, activity, or contact: the same microbial observations could be likely if a person directly touched an object, indirectly transferred material, shared an environment, or handled the object at an earlier time. A source-level association should not be overstated as evidence of a particular activity.

Until representative probability models are established, results should be reported as investigative or supportive information with explicit limitations. Qualitative phrases such as ‘consistent with’ can also be misunderstood unless the laboratory states what was compared and what alternatives were considered. Microbiome findings should be integrated with STRs, fingerprints, trace materials, scene history, and other independent evidence rather than treated as standalone identification (Figure 2; Tables 1, 2).

Implementing likelihood-based reporting in practice would require: (i) empirical estimates of within-person variability across relevant time intervals and body sites; (ii) between-person variability estimates from a relevant population database, stratified by factors that affect microbial profiles; (iii) measurement error distributions for the specific analytical pipeline; (iv) models for transfer, persistence, and background that are validated for relevant substrate and environmental conditions; and (v) a framework for combining multiple microbial features without violating independence assumptions. Until these components are established, likelihood ratios should not be offered as quantitative evidence; instead, results should be reported as investigative or supportive information with explicit limitations.

## 5 Evidence for donor association and source inference

### 5.1 Community-level individual differentiation

The earliest forensic studies demonstrated that skin bacterial communities differ among people and that traces recovered from personal objects can resemble the owner’s fingertips (Fierer et al., 2010). This finding established that skin-associated microbial transfer contains donor information. Later longitudinal and metagenomic studies showed that personal microbial features can persist over time (Franzosa et al., 2015; Oh et al., 2016). Collectively, these studies provide strong biological proof of concept.

Community-level identification is nevertheless sensitive to experimental design. Small cohorts and repeated samples per person create a favorable closed-set problem in which each donor has multiple closely related training examples. Performance typically declines when later time points, different body sites, or unseen environmental conditions are tested. Dominant taxa shared by many people may also limit discrimination. Thus, broad 16S profiles are useful for ecological comparison and screening but may be insufficient for high-confidence individualization.

Some studies report that a relatively small number of informative taxa can classify participants with high accuracy. This observation is valuable for assay design, but the selected taxa may not remain informative in another population. Reported accuracy should always be interpreted with the number of donors, sampling frequency, train-test separation, and candidate pool size. A model distinguishing 10 known volunteers is not equivalent to searching a city-scale population.

### 5.2 Targeted markers, SNPs, and high-resolution classifiers

Targeted clade-specific markers and microbial SNPs represent a more forensic-oriented strategy because they focus on variation expected to be stable and discriminating (Schmedes et al., 2018; Woerner et al., 2019; Sherier et al., 2021, 2022). Nearest-neighbor and machine-learning analyses have shown that such panels can associate samples with donors in controlled datasets. The fixed feature space may facilitate analytical validation, sensitivity studies, marker dropout assessment, and future probabilistic modeling.

Higher-resolution studies using species-level and SNP profiles or reduced-representation metagenomics have further improved donor separation (Huang et al., 2025; Li et al., 2026). Deep-learning architectures can identify interactions among many markers, but their apparent performance must be evaluated against overfitting and data leakage. Models should be compared with simpler baselines and should provide calibration or uncertainty measures. Explainability is not a substitute for validation, but it can reveal whether the model relies on biologically plausible markers or on batch-specific artifacts. At present, the evidence supports controlled donor differentiation rather than universal forensic identification. External population validation, repeated sampling over longer periods, mixture studies, low-template sensitivity, and interlaboratory reproducibility remain limited. The appropriate current wording is therefore that targeted and strain-level approaches are promising candidates for complementary human association, not that they provide an equivalent to STR individualization.

### 5.3 Body-site and biological-trace source inference

Anatomical structure creates the possibility of inferring whether a microbial trace originated from a palm, face, sebaceous region, or another skin site. Related studies have also examined microbial patterns associated with different biological traces or body fluids (Huang et al., 2024, 2025). Higher-resolution species and SNP features may improve separation when broad taxa overlap. Such information could help prioritize downstream testing or interpret how a trace was deposited.

Source inference is vulnerable to confounding by personal care, disease, contact, and the surrounding environment. A palm that has recently touched the face or a contaminated surface may carry a mixed signal. Biological fluids can also acquire skin and environmental organisms during deposition or collection. Models should therefore be evaluated on mixed and cross-contaminated specimens, and outputs should be probabilistic or tiered rather than categorical. Body-site inference can also improve donor models by ensuring that questioned and reference samples are anatomically compatible. Conversely, it can inflate person classification if all samples from each donor come from a unique site or batch. Study design must separate donor, body site, and processing effects.

## 6 Contact inference and interpersonal microbial exchange

### 6.1 Skin-to-object transfer

Microbial transfer from hands to objects is the most direct bridge between skin ecology and forensic contact inference. Studies of keyboards, glass, clothing, and touched surfaces show that donor-associated communities can be recovered after contact (Fierer et al., 2010; Procopio et al., 2021, 2024; Wang et al., 2026). The signal is influenced by donor shedding, contact intensity, substrate, and environmental background. These studies justify further development because they demonstrate recoverability under conditions where conventional DNA may be incomplete.

The central forensic limitation is that detection does not prove when, how, or during which event transfer occurred. Direct contact, secondary transfer, shared environments, and prior legitimate handling can all produce similarity. A person who frequently uses an object may leave a persistent background profile that remains after another person touches it. Conversely, a recent contact may deposit little microbial material. The common claim that the ‘last contactor’ can be identified should therefore be avoided unless a study specifically validates that proposition across realistic contact sequences. Substrate properties matter. Smooth nonporous surfaces may permit efficient swabbing but expose cells to desiccation and cleaning. Textiles retain material within fibers but also contain manufacturing, laundering, wearer, and environmental microbiota. Metal, plastic, painted wood, paper, and glass create different survival conditions. Transfer models should either be substrate-specific or demonstrate transportability across materials.

### 6.2 Persistence, decay, and secondary transfer

A deposited community can change through physical loss, DNA degradation, selective survival, microbial growth, and later contacts. Simple exponential decay is unlikely to describe all taxa or substrates. Some organisms may persist as DNA after loss of viability; others may increase under favorable humidity. Accordingly, relative-abundance changes may reflect ecological selection rather than elapsed time alone. Controlled persistence studies should include repeated sampling, environmental monitoring, and untreated background controls. They should report whether the same object is sampled destructively or repeatedly, because repeated swabbing changes the deposit. Secondary-transfer experiments should test realistic intermediates such as gloves, clothing, shared devices, and hands. The evidentiary question should be specified in advance: source association, presence of contact, recency, or order of contact require different designs.

At present, microbial traces can support a hypothesis that contact or shared exposure is plausible, but they cannot reliably determine directness or timing in complex scenes. Their value may be greatest when they generate investigative leads or corroborate independent evidence rather than when they are used alone to infer a precise interaction.

### 6.3 Interpersonal exchange and network inference

Close interpersonal contact can produce bidirectional microbial exchange, and cohabitation creates shared exposure over longer periods. In principle, comparison of multiple people and objects could reveal clusters of microbial similarity consistent with interaction. Network analysis offers an intuitive way to visualize these relationships, especially in households, workplaces, or group events. However, a similarity network is not automatically a contact network. People may share microbes because they occupy the same room, touch common objects, have similar lifestyles, or were processed in the same laboratory batch. Direction and timing are particularly difficult to infer because the recipient’s baseline and shedding rates are often unknown. A dense network can also emerge from ubiquitous taxa. Network edges should therefore represent explicitly defined and validated statistical evidence rather than arbitrary distance thresholds.

Chronological contact reconstruction remains exploratory. Abundance gradients and mixture proportions may contain temporal information in carefully controlled experiments, but robust ordering of several contacts has not been demonstrated across realistic delays, substrates, cleaning, and secondary transfer. Claims in this area should be framed as hypotheses for future validation, not operational capability.

## 7 Skin microbiome evidence in crime-scene reconstruction

### 7.1 Postmortem microbial succession

A critical distinction must be drawn between two fundamentally different forensic questions involving postmortem skin microbiology. Identifying a deceased individual using their skin microbiome is not currently feasible: after death, the skin environment changes dramatically—immune surveillance ceases, circulation stops, oxygen tension drops, moisture and temperature profiles shift, and the microbial community undergoes rapid succession that quickly erodes the donor-specific signature present during life. The postmortem skin microbiome is therefore unsuitable for matching a deceased person to a living reference profile. In contrast, estimating the postmortem interval (PMI) exploits the predictable successional changes themselves as a time-dependent signal. These two applications have different biological bases, different validation requirements, and different levels of evidentiary feasibility. PMI modeling is a credible research direction, whereas postmortem donor identification should not be pursued with current microbiome methods. The specific conditions under which each application might be feasible are: PMI estimation may be feasible within defined time windows, controlled environments, and with appropriate local calibration; postmortem identification is not supported by current evidence under any conditions.

Death alters immune control, circulation, oxygen availability, moisture, temperature, and access of internal and environmental organisms to the skin. These changes can produce time-associated succession in skin and adjacent microbial communities. Human and model-system studies have identified postmortem shifts and have used machine learning to predict PMI (Iancu et al., 2023; Johnson et al., 2016; Metcalf, 2019; Belk et al., 2018; Zhang et al., 2021; Hu et al., 2024). The general biological premise is credible: decomposition is an ecological process, and microbial communities respond to its changing substrates and conditions. The specific trajectory is not universal. Anatomical site, ambient temperature, humidity, clothing, burial, insect activity, cause of death, medical treatment, season, and geographic location can influence succession. Early postmortem skin may retain commensal taxa, while later stages increasingly reflect anaerobic and environmental organisms. Diversity may decline in some settings and increase in others as niches open. A single ratio, such as *Cutibacterium* to *Clostridium* or *Staphylococcus*, cannot be assumed to function as a universal clock.

Human cadaver studies are especially valuable but remain difficult because sample sizes are limited and metadata are heterogeneous. Some reports focus on the first hours after death, whereas others study days or burial intervals. Prediction error depends on the time window and validation scheme. A narrow error reported in a small controlled interval should not be generalized to all PMIs or environments. Models require geographically and seasonally diverse external validation before they can supplement established entomological, biochemical, and pathological indicators (Iancu et al., 2023; Johnson et al., 2016; Metcalf, 2019; Belk et al., 2018; Zhang et al., 2021; Hu et al., 2024).

### 7.2 PMI model development and validation

PMI models commonly use random forest regression or related machine-learning methods to map microbial features to elapsed time (Johnson et al., 2016; Belk et al., 2018; Zhang et al., 2021; Hu et al., 2024). Such models can accommodate nonlinear succession and interactions among taxa. They are also susceptible to learning site, season, or study-specific labels. Validation should hold out entire cadavers, collection periods, and locations rather than random aliquots from the same body.

Environmental variables should be incorporated prospectively. Accumulated degree hours or days may better represent microbial activity than clock time alone, but temperature measurement itself can be uncertain. Humidity, rainfall, burial depth, clothing, and insect access may need separate parameters. Multimodal approaches that combine microbial data with metabolites, volatile compounds, entomology, or physical observations may be more robust than a microbiome-only model (Metcalf, 2019; Hu et al., 2024). Operational reporting should emphasize prediction intervals and the validated range. A model trained on 1–7 days should not extrapolate to weeks, and a model trained in one climate should not be used in another without validation. The most defensible near-term role is complementary PMI estimation within defined conditions, not replacement of established methods. It must also be acknowledged that PMI models are susceptible to the same environmental and post-mortem confounders as other microbiome applications; prediction intervals should therefore be interpreted with caution and never as a substitute for established pathological or entomological indicators.

### 7.3 Biological-trace and environmental source attribution

Microbial profiles recovered from fingerprints, saliva residues, bloodstains, clothing, or objects may contain information about the contributing body site, individual, and environment. Comparison with background databases can help distinguish human-associated and environmental components (Huang et al., 2024, 2025; Clarke et al., 2017; Lax et al., 2014). This could be useful when human DNA is degraded, when the material is too limited for a complete STR profile, or when the investigative question concerns location rather than identity.

Environmental source attribution requires representative databases. Indoor microbiomes vary among buildings, rooms, occupants, ventilation systems, animals, and cleaning regimes. Outdoor communities vary with soil, vegetation, climate, and season. A questioned sample may combine several environments accumulated over time. Therefore, a match to a reference environment should be interpreted as relative support among considered sources, not proof that the object followed a unique route. Background sampling is critical. Investigators should collect controls from nearby surfaces, comparable untouched items, collection tools, and relevant alternative locations. Without background data, common environmental taxa can be incorrectly attributed to a suspect or object. In many cases, microbial source information will be most valuable for hypothesis generation and exclusion of implausible scenarios.

### 7.4 Spatial trajectory and activity-sequence reconstruction

The concept of reconstructing movement through a scene from layered microbial traces is scientifically attractive. An object could, in principle, acquire signatures from a household, vehicle, victim, and final scene, and mixed-source models might estimate their contributions. Geospatial visualization and network inference can then display alternative scenarios.

The present evidence does not support routine reconstruction of a unique spatial trajectory. Microbial signals are neither deposited nor retained in a simple chronological stack. Each contact has different transfer efficiency, and later events may add, remove, or amplify organisms. Similar environments can share taxa, while an individual’s skin can carry environmental material away from its source. Deconvolution is particularly unstable when reference environments are incompletely sampled. Future validation should use blinded mock scenes with pre-defined competing scenarios, multiple actors, realistic cleaning and delays, and independent analysts. Models should compare the likelihood of specific alternatives rather than generating a visually persuasive but uncalibrated heat map. Until then, spatial–temporal reconstruction should be presented as an exploratory research direction.

## 8 Evidence maturity, validation, and standardization

### 8.1 Current maturity across applications

The evidence is not equally mature across forensic questions. To provide a clearer framework for readers and practitioners, we classify applications into three tiers:

Established (proof-of-concept level): Inter-individual skin microbial variation and short-term transfer to touched objects are well-supported biological observations. Controlled donor classification using targeted or high-resolution markers is promising but lacks the population scale and external validation of routine identification methods.

Emerging (broader validation needed): Body-site and biological-trace source inference have growing empirical support (Huang et al., 2024; Huang et al., 2025). PMI modeling has a credible ecological basis but remains context-dependent and requires geographically diverse external validation (Iancu et al., 2023; Johnson et al., 2016; Metcalf, 2019; Belk et al., 2018; Zhang et al., 2021; Hu et al., 2024).

Exploratory (conceptual only): Contact order reconstruction, interpersonal network direction inference, and full crime-scene trajectory reconstruction remain in the conceptual and simulation phase. These should be explicitly framed as research directions rather than capabilities.

This distinction matters because graphical summaries can make all stages appear equally operational. A conceptual workflow should therefore display uncertainty and validation gaps. In the revised figures, established findings, emerging methods, and exploratory reconstruction tasks are separated rather than merged into a single seamless forensic pipeline (Figure 3; Table 3).

**Figure 3:**
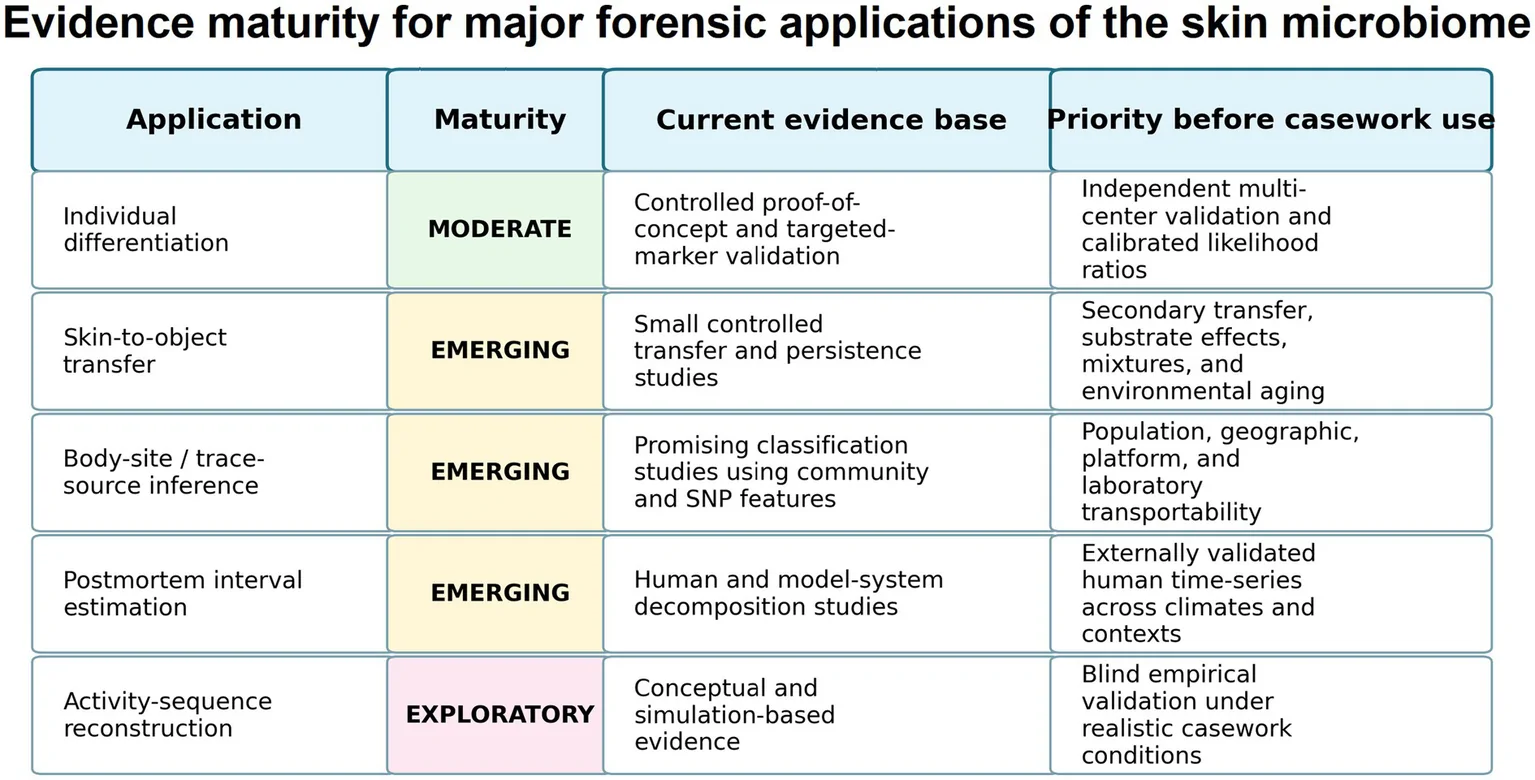
Evidence maturity across major forensic applications of the skin microbiome. Individual differentiation and controlled touch transfer have the strongest proof-of-concept basis; body-site inference and PMI modeling are emerging; chronological contact and spatial trajectory reconstruction remain exploratory. The figure is a conceptual schematic and does not contain experimental data.

**Table 3:** Minimum validation framework before operational use.

Domain	Minimum requirement	Recommended evidence before operational use
Pre-analytical	Specified swab, area, pressure, site, substrate, storage, and chain of custody	Field blanks, scene metadata, substrate and delay studies
Extraction	Combined lysis appropriate to target; randomized batches; biomass quantification	Extraction blanks, positive controls, replicates, reagent-lot assessment
Sequencing or assay	Defined targets, depth, analytical sensitivity and specificity	Reference materials, marker dropout studies, run controls
Bioinformatics	Versioned pipeline, contaminant filtering, batch assessment	Locked code and databases; reproducible analysis package
Model development	Donor-level separation, nested tuning, class-balance reporting	No replicate leakage; pre-specified metrics and inconclusive class
External validation	New donors, time points, substrates, populations, and laboratories	False-match and false-non-match rates; open-set and mixture testing
Interpretation	Explicit propositions, uncertainty, and limitations	Likelihood-based framework or clearly limited investigative reporting
Quality assurance	SOPs, proficiency testing, corrective actions, audit trail	Multicenter exercises and independent scrutiny

### 8.2 Minimum analytical validation

Before operational use, laboratories need validated standard operating procedures for collection, storage, extraction, amplification, sequencing, contamination control, and bioinformatics. Analytical sensitivity should define the minimum microbial biomass or read depth required for reliable results. Specificity should address cross-reactivity and environmental background. Precision should be evaluated through repeat extraction, library preparation, and sequencing. Mixture, inhibition, degradation, substrate, and environmental studies should use conditions relevant to casework.

Reference materials are a major gap. Defined mock communities can monitor taxonomic recovery, but they do not fully represent low-biomass skin traces. Stable cell mixtures, synthetic microbial DNA, and characterized donor-derived materials may be needed for different validation stages. Proficiency testing and interlaboratory exercises should determine whether the same evidence produces comparable results across institutions (Ruuskanen et al., 2022; Balacco et al., 2025; Knight et al., 2018; Sinha et al., 2017).

### 8.3 Population, temporal, and field validation

A forensic method must perform in the population to which it is applied. Reference datasets should include geographic, demographic, occupational, health, and lifestyle diversity without treating sensitive attributes as deterministic. Repeated sampling is needed to estimate within-person variation, and unrelated persons should be sampled using the same sites and methods to estimate between-person variation. Database growth studies should determine how performance changes as more candidate donors are added. Field validation should use evidence collected by trained scene personnel, realistic delays, common surfaces, environmental background, and routine laboratory constraints. Blinded studies are preferable to analyses in which investigators know the expected donor. Casework-like studies must also evaluate inconclusive outcomes and the consequences of missing reference sources (Figure 3; Table 3).

### 8.4 Reporting standards and reproducibility

Published studies should report sampling area, pressure or passes, storage, extraction kit and lysis, control results, primer regions or target loci, sequencing depth, software versions, database versions, feature-filtering rules, and all train-test separation decisions. Raw sequence data and analysis code should be shared when consent and privacy allow. When open release is inappropriate, controlled-access repositories or synthetic benchmark datasets can support reproducibility.

Performance claims should state the exact task: same-session classification, later-sample recognition, closed-set donor assignment, open-set recognition, source inference, or activity-level evaluation. Accuracy without these details is of limited forensic meaning. Negative and inconclusive results should be reported because publication bias toward high-performing classifiers creates an unrealistically favorable literature (Figure 3; Table 3).

## 9 Ethical, privacy, and legal considerations

### 9.1 Privacy and data governance

Microbial evidence differs from STR profiling in the breadth of incidental information it may contain. Community and metagenomic data can reflect health, medication, diet, occupation, household sharing, travel, animal contact, and environmental exposure. Shotgun data may also contain human reads. These features create risks of function creep, in which a sample collected for source comparison is later used to infer unrelated personal characteristics (McGuire et al., 2008).

Governance should follow data-minimization principles. Laboratories should collect and retain only the information needed for the stated forensic purpose, separate human and microbial reads, restrict secondary analysis, and define retention periods. Consent and legal authority for reference databases require careful consideration. Population sampling should avoid stigmatizing communities or presenting probabilistic associations as biological determinism. Since microbiomes are shared through households and contact, one person’s profile may indirectly reveal information about others who never provided a sample. This relational aspect strengthens the need for controlled access, transparent reporting, and independent oversight.

### 9.2 Legal admissibility and evidentiary weight

Courtroom use requires more than peer-reviewed proof of concept. Under admissibility frameworks such as Daubert, relevant considerations include empirical testing, known or potential error rates, standards controlling operation, peer review, and acceptance within the scientific community. Skin microbiome evidence currently lacks standardized casework protocols, large-scale error-rate studies, and broadly accepted probabilistic interpretation. These gaps do not prevent research or carefully limited investigative use, but they constrain claims of identification.

Activity-level propositions are especially challenging. A microbial association between a person and an object may be compatible with direct contact, secondary transfer, prior legitimate handling, or shared environment. The expert should distinguish source-level and activity-level conclusions and avoid implying that a source association establishes criminal conduct. Reports should explain alternative mechanisms and should not use categorical language unsupported by validation.

Laboratory accreditation alone will not resolve these interpretive limitations. Validity depends on the method, data, propositions, and case context. Independent review, proficiency testing, transparent software, and access to underlying validation are necessary for meaningful scrutiny.

### 9.3 Responsible communication

Microbiome results are visually compelling. Heat maps, clustering trees, and network diagrams can create an impression of precision that exceeds the underlying evidence. Figures used in manuscripts or court should distinguish measured data from conceptual illustration, provide legends and uncertainty, and avoid decorative elements that resemble empirical results.

Terminology also matters. “Contact inference” is preferable to ‘contact tracing’ in this context because the latter has an established public-health meaning. ‘Association’ or ‘support’ is generally more appropriate than ‘identification’ when population probabilities and error rates are unavailable. Clear language protects both scientific credibility and legal fairness.

## 10 Research priorities and implementation roadmap

The next phase of forensic skin microbiome research should prioritize validation rather than continually adding more complex classifiers. First, multicenter studies should use harmonized collection and laboratory protocols while intentionally varying geography, climate, body site, substrate, and time. Second, marker panels and metagenomic models should be locked before external testing. Third, open-set and mixture evaluation should become routine. Fourth, study reports should provide false-match and false-non-match rates, calibration, and inconclusive thresholds rather than only overall accuracy. Reference databases must be built with representative populations and environments. A useful resource would include longitudinal donor samples, multiple anatomical sites, common substrates, controlled transfer histories, indoor and outdoor backgrounds, and standardized metadata. Governance should be designed at the outset, with controlled access for sensitive data and public release of non-identifying benchmark materials.

Portable long-read sequencing may eventually support rapid field screening, but portability does not eliminate the need for contamination control and interpretation. On-site sequencing can amplify scene-associated background and may complicate chain of custody if data processing is not standardized. Near-term deployment is more realistic for triage or research than for definitive identification. Multi-omics approaches could combine microbial taxa with metabolites, proteins, volatile compounds, or host-independent environmental markers. Such integration may improve PMI or source inference because different signals respond to different confounders. It also increases analytical complexity and privacy risk. Added features should be retained only when they improve external validation and calibrated decision-making. A staged implementation pathway is therefore appropriate. Stage 1 comprises standardized research collection and interlaboratory reproducibility. Stage 2 includes locked assays and blinded external validation. Stage 3 evaluates realistic mock cases, mixtures, transfer, and competing propositions. Stage 4 consists of limited investigative use with explicit reporting boundaries. Court-oriented evidentiary use should be considered only after error rates, population models, quality assurance, and independent scrutiny are established.

## 11 Limitations of this review

This review has several inherent limitations that should be considered when interpreting its findings. First, as a narrative rather than systematic review, it does not employ a comprehensive, reproducible search strategy across multiple databases; PubMed/MEDLINE served as the primary source, and while backward citation searching was performed, relevant studies indexed only in Scopus, Web of Science, relevant studies indexed only in Scopus or Web of Science may have been omitted. Second, the heterogeneity of study designs, outcome measures, and validation approaches precluded quantitative synthesis or meta-analysis; our maturity classifications (established/emerging/exploratory) are qualitative judgments based on the authors’ assessment of the evidence base rather than a formal scoring system. Third, the review focuses primarily on bacterial markers, reflecting the current literature; fungal, viral, and multi-kingdom approaches are discussed but may be underrepresented relative to their potential future importance. Fourth, the legal and ethical analysis is general and jurisdiction-agnostic; admissibility standards, privacy laws, and regulatory frameworks vary substantially across countries, and specific legal requirements are not addressed in detail. Fifth, the review does not provide primary empirical data or original validation; it synthesizes existing studies and identifies gaps but does not fill them. We encourage readers to consult the primary literature for detailed methodological and population-specific findings. These limitations are inherent to the review format and scope; they do not undermine the critical synthesis but should inform the weight placed on specific conclusions.

## 12 Conclusion

The realistic utility of skin microbiome evidence in current forensic practice can be summarized candidly as follows: it is viable for generating investigative leads by suggesting possible associations between persons, objects, or locations; for excluding improbable scenarios when a questioned profile is clearly dissimilar to a reference; for suggesting the likely anatomical origin of a trace; and for corroborating other evidence when consistent with independent findings. It is not reliable for definitive human identification comparable to STR profiling, nor for reconstructing specific contact events in complex scenes. This pragmatic assessment informs the detailed conclusions that follow.

The skin microbiome offers a biologically plausible and increasingly measurable source of forensic information. Individual-specific variation, temporal persistence of some strain-level features, and transfer to contacted surfaces support applications in donor differentiation and contact-related investigation. Targeted microbial markers, SNPs, full-length sequencing, metagenomics, and machine learning have strengthened analytical resolution. However, the observed profile is an ecological trace rather than a fixed identifier. It is shaped by body site, time, environment, substrate, contamination, transfer history, and laboratory workflow.

The strongest current evidence supports proof-of-concept individual differentiation and controlled skin-to-object transfer. Body-site and biological-trace inference are emerging, while PMI estimation remains condition-dependent and requires broader validation. Chronological contact reconstruction and spatial trajectory inference should remain explicitly exploratory. Across all applications, classification accuracy must not be equated with forensic evidentiary weight. Skin microbiome profiling should therefore complement, not replace, human DNA, fingerprints, and conventional trace evidence. Its responsible translation requires harmonized protocols, low-biomass controls, representative longitudinal databases, donor-level and external validation, calibrated uncertainty, privacy safeguards, and legal interpretation based on competing propositions. Meeting these requirements would allow the field to progress from visually persuasive demonstrations toward transparent and defensible forensic science.

Thus, across the four application domains evaluated, the classification is clear: individual differentiation and controlled touch transfer are established at the proof-of-concept level; body-site and trace-source inference are emerging; PMI estimation is emerging but condition-dependent; and chronological/spatial reconstruction remains exploratory.
